# Single‐cell transcriptome analysis of tumor‐infiltrating B cells reveals their clinical implications in non‐small cell lung cancer

**DOI:** 10.1111/1759-7714.13719

**Published:** 2020-10-29

**Authors:** Xia‐Yao Diao, Fang Wang

**Affiliations:** ^1^ Department of Urology Sun Yat‐sen Memorial Hospital Guangzhou China; ^2^ State Key Laboratory of Oncology in South China Collaborative Innovation Center for Cancer Medicine, Sun Yat‐sen University Cancer Center Guangzhou China; ^3^ Department of Molecular Diagnostics Sun Yat‐sen University Cancer Center Guangzhou China

Lung cancer is the most common cancer in 2020, accounting for 25% of all cancer‐related deaths in the world.^1^ China is one of the top 20 countries with the highest incidence and mortality of lung cancer.^2^ The main subtype of lung cancer is non‐small cell lung cancer (NSCLC). With the development of various treatments, the clinical outcome of NSCLC has been significantly improved; however, the prognosis of advanced NSCLC is still poor.^3^ Increasing evidence suggests that immunotherapy is a promising therapeutic strategy for advanced NSCLC.^4^ The efficacy of immunotherapy mainly depends on the characteristics of the tumor microenvironment.^5^ Identification and structural characterization of tumor microenvironment landscape and the interactions between different cell types is a key step to improve the efficacy of tumor immunotherapy.

T cells and B cells are the major populations of immune cells, which play a vital role in tumor initiation and progression. Recently, T cells have become the most attractive therapeutic target of immune checkpoint inhibitors in immunotherapy, but only 20%–25% of unscreened NSCLC patients can respond to immune checkpoint inhibitors.^6^, ^7^, ^8^, ^9^ As an important component of adaptive immunity, tumor‐infiltrating B cells are potential targets of cancer immunotherapy. However, the role of tumor‐infiltrating B cells in NSCLC immunotherapy is reported to be inconsistent.^10^ For example, a previous study reported that using anti‐IgM antibodies to deplete B cells can inhibit tumor growth in mouse models, indicating that B cells play a critical role in promoting cancer cell progression.^11^ On the contrary, another study reported using anti‐CD20 antibodies to deplete B cells enhanced the progression of mouse melanoma, which reveals the potential antitumor activity of B cells.^12^ This double‐edge effect may be caused by the different proportions of different B cell subtypes and their interaction with other cell types in the tumor microenvironment. However, the subtypes of B cells exist in NSCLC tissues, and the molecular mechanisms by which they interact with cancer cells and other stromal cells are still unknown. Therefore, there is an urgent need to characterize all immune cell types in the tumor microenvironment, including B cells, with single‐cell resolution.

Single‐cell transcriptome analysis is a method that can comprehensively characterize cell populations with high resolution. The immune contexture defined by this method has been proven to be used for cancer prognosis prediction and immunotherapy guidance.^13^ However, this method is now mainly used to analyze T cells in the tumor microenvironment, while tumor‐infiltrating B cells have been largely ignored. In a study recently published in *Genome Biology*, titled “Single‐cell transcriptome and antigen‐immunoglobin analysis reveals the diversity of B cells in non‐small cell lung cancer”,^14^ Chen *et al*. from China examined the tumor‐infiltrating B cell profiles from NSCLC patients by single‐cell RNA‐sequencing and analyzed the relationship between the sequencing results and the prognosis of participants.

A total of 115 545 cells in fresh tumor tissues from 11 NSCLC patients were examined by single‐cell transcriptome analysis. Based on the results, the authors identified 22 distinct cell clusters by using known specific markers, including tumor cells, monocytes, lymphocytes, and epidermal cells. B cells could be divided into two major cell clusters: B cell‐cluster 1 express naïve B cell markers (CD19, CD20, CD22, CD83, and TCL1A) and B cell‐cluster 2 express plasma B cell markers (CD38, TNFRSF17, and IGHG1/IGHG4). The ratio of each cell cluster were detected in tumor tissues of different patients. The results revealed that the percentage of naïve‐like B cells in stage I was significantly higher when compared with stage III. Additionally, NSCLC patients with a high percentage of naïve‐like B cells in tumor tissues had a better prognosis. This observation was further confirmed in a cohort containing 164 NSCLC specimens. The in vitro experiments show that coculture with naïve‐like B cells can significantly inhibit the proliferation of lung cancer cell lines. In contrast, plasma‐like B cells from different stages of tumor tissues had different effects on lung cancer cell lines. The plasma‐like B cells from stage I tumor tissues suppressed the proliferation of lung cancer cells, while those from stage III tumor tissues enhanced cancer cell proliferation. By analyzing the conditioned medium of plasma‐like B cells from different stages of NSCLC, the authors found that plasma‐like B cells from different stages had different effects on tumor cells and these effects mainly depends on the IgGs they secreted. In order to illustrate the biological functions of these IgGs, the authors identified their target proteins through immunoprecipitation assay. From the results they found that the assembly polypeptide 2 (AP2) complex can deliver IgGs into tumor cells; in addition, the IgGs that entered tumor cells can recognize the targets through their Fab domains and then interact with tripartite motif‐containing 21 (TRIM21) through their Fc domains to trigger RHOC ubiquitin‐mediated degradation.

This study demonstrated that B cells can be divided into many different functional subgroups in NSCLC. B cell‐associated immunotherapy should target a single or multiple unique subgroups of B cells, rather than all B cells. Additionally, the single‐cell transcriptome data of this study provides a reference gene expression landscape of distinct subtype of tumor‐infiltrating B cells compared to their normal counterparts from peripheral blood. Therefore, exploring the unique role of the specifically expressed genes of tumor‐infiltrating B cells in NSCLC development is another direction for future research. Moreover, this study also discovered a new molecular mechanism of antibody endocytosis in NSCLC cells. AP2 complexes are the major players in clathrin‐mediated endocytosis. This study found that AP2 complex is directly involved in the endocytosis of IgGs in NSCLC cells. We all clearly know how IgGs target extracellular proteins, but how they target cancer cells is still a mystery. This study has uncovered the facts. It demonstrated that a cytosolic Fc receptor (TRIM21) can directly bind to IgGs and deliver the ubiquitin proteasome to targets that recognize Fab. Indeed, a recent study found that the antibody‐TRIM21‐ubiquitin proteasome is a tool that could be used to degrade specific targets.^15^ Another important finding of this study is that Rhoc antibodies can be delivered into NSCLC cells by AP2 complexes and then mediate the degradation of Rhoc proteins in NSCLC cells, leading to the inhibition of tumor progression. This finding reveals the potential role of IgG in antitumor response.

Overall, for the first time, this study established the versatile function of B cell subtypes and immunoglobulins in regulating NSCLC development, highlighting the potential clinical significance of targeting B cell subgroups in the NSCLC microenvironment, and provides a solid foundation for the development of new immunotherapy strategies in the future.

## Disclosure

The authors declare that there are no conflict of interests.
